# CorA_2_-mediated magnesium transport is essential for stress adaptation and virulence of *Streptococcus agalactiae*

**DOI:** 10.1186/s13567-026-01757-3

**Published:** 2026-04-21

**Authors:** Jiayi Liu, Fengyang Li, Yong-An Zhang, Hui Zeng

**Affiliations:** 1 Hubei Hongshan Laboratory, College of Fisheries, Huazhong Agricultural University, Wuhan, 430070 China; 2 Hubei Jiangxia Laboratory, Wuhan, 430200 China

**Keywords:** *Streptococcus agalactiae*, CorA_2_, magnesium transport, virulence, tilapia

## Abstract

Magnesium (Mg^2+^) homeostasis is critical for bacterial survival and virulence. *Streptococcus agalactiae* is a major piscine and mammalian pathogen, yet the molecular mechanisms governing its Mg^2+^ acquisition remain undefined. Here, we identify and characterize CorA_2_ as an essential Mg^2+^ transporter in a hypervirulent *S. agalactiae* strain HN016. Through biochemical and genetic assays including a *corA*_*2*_ deletion mutant (Δ*corA*_*2*_), a complemented strain, and structure-guided point mutants (GGGG gain of function and D206K loss of function), we demonstrate that CorA_2_ is essential for Mg^2+^ uptake. CorA_2_ deficiency severely impaired bacterial growth under Mg^2+^ limitation and heightened susceptibility to host-mimicking stresses, including oxidative stress, acid stress, nitrosative stress, and metal ion toxicity. The Δ*corA*_*2*_ mutant was markedly compromised in resisting phagocytosis and surviving within macrophages. Intriguingly, while the mutant exhibited enhanced adhesion to and invasion of host cells, its capacity to inflict damage was drastically reduced. In a tilapia infection model, the Δ*corA*_*2*_ strain showed severe attenuation, with significantly reduced mortality (53.3% versus 93.3% in WT) and systemic bacterial burden. Our findings establish that CorA_2_-mediated magnesium homeostasis is essential for *S. agalactiae* to overcome host immune defenses and establish a systemic infection, highlighting CorA_2_ as a potential target for developing novel anti-infective strategies against this pathogen.

**Supplementary Information:**

The online version contains supplementary material available at 10.1186/s13567-026-01757-3.

## Introduction

Streptococcosis represents a major threat to global food security, causing substantial economic losses due to mass mortality in farmed fish [1]. Among the causative agents, *Streptococcus agalactiae*, also named group B *Streptococcus*, has emerged as a predominant pathogen since 2008, particularly in the tilapia industry [2]. Compounding its veterinary impact, *S. agalactiae* is also a significant zoonotic pathogen, capable of causing serious infections such as neonatal sepsis, endocarditis, meningitis, and pneumonia in the elderly and pregnant women [3–6]. The control of streptococcosis caused by *S. agalactiae* remains heavily dependent on antibiotics, a practice that fosters antimicrobial resistance [7]. Understanding the molecular mechanisms of *S. agalactiae* pathogenesis is therefore crucial for developing novel, effective anti-infective strategies to combat this disease.

A critical determinant of bacterial pathogenesis is the ability to acquire essential nutrients within the hostile host environment. Magnesium (Mg^2+^) is an essential metal ion for pathogenic bacteria, acting as a cofactor in numerous enzymatic reactions [8]. Consequently, Mg^2+^ restriction has been recognized as a key component of nutritional immunity [9, 10]. This is exemplified by macrophages, which impair *Salmonella enterica* serovar Typhimurium survival by sequestering intracellular Mg^2+^ via the SLC11A1 transporter. The physiological relevance of this battle is confirmed by the attenuated virulence of *S*. Typhimurium in deficient (*Slc11a1*−/−) compared with wild-type (*Slc11a1*+/+) mice [11]. These discoveries highlight the battlefield over this essential metal at the host–pathogen interface.

Prokaryotes carry three principal classes of Mg^2+^ transport systems: CorA, MgtA/B, and MgtE. Among these, the CorA family serves as an essential Mg^2+^ transporter and is widely conserved across bacteria and archaea, underscoring its fundamental physiological role [12]. The critical role of CorA in bacterial physiology is evidenced by its necessity for virulence in pathogens as diverse as *Pectobacterium versatile* [13] and *Haemophilus influenza* [14]. Notably, in *S*. Typhimurium, which encodes the MgtA/B transporters, mutation of *corA* alone is sufficient to attenuate virulence and cause other defects [15], highlighting its critical function in infection across bacterial species. Structural studies have elucidated the conserved architecture of CorA family transporters, primarily through the determination of the full-length crystal structure of *Thermotoga maritima* CorA [16]. Key mechanistic features include a GMN motif that acts as a selectivity filter for Mg^2+^ and conserved intracellular acidic residues that form metal-binding sites (e.g., the M1 site) [17]. These sites function as intrinsic Mg^2+^ sensors; cation binding stabilizes the channel in a symmetric, closed conformation, thereby mediating Mg^2+^-dependent gating [17]. Although site-directed mutagenesis has confirmed the functional importance of these residues [18, 19], a direct mechanistic understanding of how CorA gating regulates virulence in specific pathogens is still lacking.

Magnesium homeostasis is fundamental to cellular physiology, however, the key transporter(s) responsible for Mg^2+^ acquisition in *S. agalactiae* remain unidentified. To address this gap, genomic analysis of *S. agalactiae* strain HN016, a hypervirulent isolate [20], identified two putative magnesium transporters: CorA (SAHN016_02720) and a second homolog SAHN016_04670, designated CorA_2_. To elucidate the functional characteristics of these two homologs, we conducted systematic bioinformatic analyses. Sequence alignment revealed that both CorA and CorA_2_ share identical sequence identity (21.26%) with the well-characterized TmCorA (Additional file 1). Further homology modeling and structural comparison demonstrated that CorA_2_ exhibits significantly higher structural similarity to TmCorA (root-mean-square difference (RMSD)RMSD = 3.809 Å) than CorA does (RMSD = 10.148 Å), suggesting that CorA_2_ may retain a more canonical CorA family fold (Additional file 1). On the basis of this structural evidence that CorA_2_ displays higher structural conservation, we selected it as the primary focus of this study. To investigate the specific role of CorA_2_, we employed a structure-guided approach, constructing a targeted deletion mutant (Δ*corA*_*2*_), a complemented strain, and two point mutants: a putative gain-of-function mutant (with a GGGG insertion designed to force channel opening) and a loss-of-function mutant (D206K, aimed at disrupting a conserved Mg^2+^-binding site). We demonstrate that CorA_2_ is essential for bacterial growth under Mg^2+^ limitation and for resistance to host-mimicking stresses, including oxidative stress, acid stress, nitrosative stress, and metal ion toxicity. Crucially, CorA_2_-mediated Mg^2+^ homeostasis is vital for overcoming phagocytic defenses and establishing systemic infection in a tilapia model. Our findings establish CorA_2_ as a critical virulence factor, providing a mechanistic foundation for targeting magnesium uptake as a novel strategy to control streptococcosis.

## Materials and methods

### Bacterial strains, cell lines, and culture conditions

*S. agalactiae* wild-type (WT) HN016 and mutant strains were routinely cultured in Todd–Hewitt broth (THB, Hopebio, China) or on THB agar plates at 37 °C as previously described [21]. For plasmid propagation, *Escherichia coli* DH5α was cultured in Luria–Bertani (LB, Hopebio, China) medium at 37 °C with shaking. When required, spectinomycin was added to the medium at a final concentration of 100 µg/mL. RAW264.7 murine macrophage-like cells were maintained under standard conditions: Dulbecco’s modified Eagle medium (DMEM; Gibco, USA) supplemented with 10% fetal bovine serum (FBS) and 1% penicillin/streptomycin, at 37 °C in a humidified 5% CO_2_ atmosphere. The tilapia brain (TiB) cell line, a kind gift from professor Weiwei Zeng (Foshan University) [22], was maintained in DMEM medium containing 10% FBS at 28 °C without 5% CO_2_.

### Construction of mutant and complementation strains

The *corA*_*2*_ deletion and genetic complementation were generated using the allelic exchange vector pSET4s and shuttle vector pSET2, respectively, as previously described [23]. The *corA*_*2*_ deletion mutant was constructed by polymerase chain reaction (PCR)-amplifying its flanking regions and fusing them through overlap extension PCR. The resulting fragment was assembled into the temperature-sensitive shuttle vector pSET4s by homologous recombination, yielding the knockout plasmid pSET4s-Δ*corA*_*2*_. This plasmid was electroporated into electrocompetent HN016 cells. Transformants were selected on THB agar containing spectinomycin (100 µg/mL) at the permissive temperature (28 °C). To promote double-crossover homologous recombination, positive colonies were serially passaged in antibiotic-free THB broth at the non-permissive temperature (37 °C). Colonies that lost the plasmid were screened by replica plating on THB broth with and without spectinomycin. Putative mutants, which grew only on antibiotic-free THB agar plates, were verified by PCR using primers flanking the deletion site and internal to the *corA*_*2*_ gene to ensure the absence of *corA*_*2*_. The *corA*_*2*_ deletion was finally confirmed by DNA sequencing, and the mutant strain was designated Δ*corA*_*2*_. For genetic complementation, the full-length *corA*_*2*_ was amplified from WT genomic DNA and assembled into the *E. coli*–*Streptococcus* shuttle vector pSET2 by homologous recombination, resulting in plasmid pSET2-CΔ*corA*_*2*_. This plasmid was introduced into the Δ*corA*_*2*_ mutant strain by electroporation. The complemented strain, selected on THB agar with spectinomycin (100 µg/mL), was designated CΔ*corA*_*2*_. Site-specific mutations were introduced into the *corA*_*2*_ gene on the plasmid pSET2-CΔ*corA*_*2*_. To generate the GGGG mutant, a flexible linker encoding four glycine (Gly) residues was inserted into CorA_2_ at the junction between threonine (Thr) 155 and threonine (Thr) 156 within the α5 and α6 helices. The D206K mutant was created by introducing a point mutation resulting in an aspartate-to-lysine substitution at position 206. The respective recombinant plasmids, pSET2-*corA*_*2*_-GGGG and pSET2-*corA*_*2*_-D206K, were electroporated into the Δ*corA*_*2*_ mutant strain. The resulting isogenic strains, selected on spectinomycin-containing THB agar, were designated GGGG and D206K, respectively. All plasmid constructs and mutant strains were verified by PCR, restriction enzyme digestion, and DNA sequencing.

### Survival index in serum

The survival index in serum assay was adapted from a previously described method [24]. Briefly, blood was collected from tilapia anesthetized with ethyl 3-aminobenzoate methanesulfonate (MS-222; cat. no. HY-W011777, MedChemExpress) at a concentration of 500 mg/L. After allowing blood to coagulate overnight at 4 °C, the sample was centrifuged at 3500 × *g* for 10 min to separate the serum fraction. The clarified serum was then aliquoted and stored at −80 °C for later use. Approximately 1 × 10^3^ colony-forming units (CFU) of mid-exponential phase bacteria in 100 μL of phosphate buffered saline (PBS, pH7.0) were mixed with an equal volume of the prepared serum, following by incubating at 28 °C for 2 h. After incubation, serial dilutions were plated onto THB agar to quantify viable bacteria. The survival index was calculated as follows: (CFU from serum-treated group/CFU from PBS-treated control group). A minimum of three independent biological replicates were included for each experiment. Of note, the inoculum size of 1 × 10^3^ CFU used here is within the range commonly employed in the field for such assays [24].

### Measurement of intracellular magnesium concentration

Intracellular Mg^2+^ levels were quantified using the fluorescent Mg^2+^ indicator Mag-Fluo-4 AM (Cat. MX4544, Maokangbio, China). Cultures of *S. agalactiae* strains were grown at 37 °C in THB until they reached the mid-exponential phase. After harvesting by centrifugation (6000 × *g*, 5 min), bacterial cells were washed three times with PBS and then resuspended in PBS to a standardized optical density at 600 nm (OD_600_) of 1.0. CFU counting confirmed that cell numbers were equivalent across strains at OD_600_ = 1.0. The cell suspension was then loaded with 1 µM Mag-Fluo-4 AM and incubated at 37 °C for 30 min in the dark. Following incubation, the cells were pelleted and washed three times with PBS to thoroughly remove extracellular dye. The final cell pellet was resuspended in PBS and transferred to a black, clear-bottom 96-well microplate (cat. no. 3601, Corning). Fluorescence intensity (excitation/emission: 488/516 nm) was measured using a BioTek Synergy HTX microplate reader. To confirm that fluorescence differences reflected intracellular Mg^2+^ content rather than dye loading efficiency, a parallel validation was performed: an equal aliquot of stained cells was lysed by sonication for 15 min, followed by addition of exogenous MgCl_2_ at final concentrations of 5, 10, and 15 mM to saturate the dye. At 5 mM Mg^2+^ supplementation, slight differences in maximal fluorescence were still observed among strains; however, at 10 mM and 15 mM Mg^2+^, no significant variation was detected among strains (Additional file 2), verifying that dye loading efficiency and membrane permeability were consistent across all experimental groups under saturating conditions. All measurements were performed in at least three independent biological replicates, with each sample assayed in technical triplicate.

### Growth curves

To evaluate growth under different magnesium conditions, we monitored the OD_600_ of *S. agalactiae* cultures. Strains were first grown in THB at 37 °C to mid-exponential phase (OD_600_ ≈ 1.0). These cultures were then diluted 1:50 into 5 mL of fresh chemically defined medium [25] supplemented with or without 5 mM Mg^2+^ (as MgSO_4_) (Table 1). The 5 mM Mg^2+^ concentration was selected not because it mimics physical conditions, but on the basis of preliminary dose–response experiments (0, 1, and 5 mM), as it provided optimal discriminatory power to resolve strain-specific differences in magnesium dependent growth. THB was used as a nutrient-rich control. Aliquots (200 µL) of each bacterial suspension were transferred in triplicate to a sterile, flat-bottom 96-well microplate (cat. no. 3585, Corning). The plate was then incubated at 37 °C in a BioTek Synergy HTX microplate reader, and the OD_600_ was automatically measured every 30 min over a 24-h period. The growth curves were plotted as the value of OD_600_ against the time.

### Assessment of stress tolerance to phagosome-mimicking conditions

The tolerance of *S. agalactiae* strains to phagosome-mimicking stresses was evaluated as previously described [26], with modifications. Bacteria were cultivated to reach a target optical density (OD_600_) of 6.5. Following centrifugation, bacterial cells were subjected to three washes with PBS and finally resuspended in 0.1 M PBS (pH 7.5) to achieve a density of 1 × 10^6^ CFU/mL. Aliquots (200 µL) of the bacterial suspension were then exposed to the following stressors for 1 h at 37 °C: 5 mM hydrogen peroxide in sodium phosphate buffer (pH 7.5, oxidative stress), 10 mM sodium nitrite (NaNO_2_) in sodium phosphate buffer (pH 4.5, acid nitrosative stress), 0.25 mM copper chloride (CuCl_2_) in sodium phosphate buffer (pH 7.5, metal ion toxicity), 100 µg/mL lysozyme in sodium phosphate buffer (pH 7.5, enzymatic challenge), sodium phosphate buffer alone (pH 4.5, acidic stress), and sodium phosphate buffer alone (pH 7.5, control). Following incubation, viable bacteria were enumerated by performing tenfold serial dilutions in PBS and plating on THB agar. The survival rate was expressed as the percentage of CFU recovered from the stressed sample compared with the untreated control. A minimum of three independent biological replicates were included for each experiment.

### Intracellular survival assay in macrophages

The intracellular survival of *S. agalactiae* strains within murine macrophage-like RAW 264.7 cells was assessed as previously described [26], with minor modifications. Prior to infection, RAW 264.7 cells were plated in 24-well plates at 5 × 10^5^ cells per well and grown to confluence. Meanwhile, mid-exponential phase *S. agalactiae* cultures were processed: after collection via centrifugation, cells were washed three times with PBS and resuspended in DMEM. The bacterial concentration was adjusted to 5 × 10^7^ CFU/mL on the basis of plate counts. Macrophage monolayers were washed twice with PBS. To initiate synchronized infection at a multiplicity of infection (MOI) of 10, wells containing cells received 900 µL of DMEM (10% FBS) and 100 µL of bacteria, after which the plate was centrifuged (800 × *g*, 10 min) and incubated (37 °C, 5% CO_2_, 1 h) to enable phagocytosis. Following incubation, the supernatant was aspirated, and monolayers were gently washed three times with PBS to remove non-internalized bacteria. To eliminate extracellular bacteria, each well was treated with 1 mL of DMEM supplemented with penicillin (5 µg/mL) and gentamicin (100 µg/mL) for 1 h. To validate the efficacy of this antibiotic regimen against all strains used in this study, a *S. agalactiae* suspension was diluted to approximately 5 × 10^6^ CFU/mL, and penicillin and gentamicin were added to final concentrations of 5 µg/mL and 100 µg/mL, respectively. The mixture was incubated at 37 °C with 5% CO_2_ for 1 h, after which 100 µL was plated onto antibiotic-free THB agar. Following 24 h of incubation at 37 °C, no colony growth was observed in the antibiotic treated group, whereas colonies grew abundantly in the untreated control group (Additional file 3). The time point immediately after this antibiotic kill step was designated as 0 h. At the indicated time points (0, 4, 8, 12, and 24 h post-treatment), wells underwent three washes with PBS. Macrophages were lysed by adding 1 mL of sterile ultrapure water per well, followed by a 10-min incubation at room temperature and vigorous pipetting. Viable counts of intracellular bacteria were obtained by performing tenfold serial dilutions of the lysates in PBS and plating them on THB agar. The anti-phagocytic ability of the strains was calculated as follows: (1 − CFU/mL at 0 h/CFU/mL in the initial inoculum) × 100%. The survival rate was calculated as follows: (CFU/mL at time point/CFU/mL at 0 h) × 100%. To visually assess phagocytic uptake, confocal microscopy was performed. Bacterial cells at mid-exponential phase were collected by centrifugation (5000 × *g*, 5 min), washed three times with PBS, and resuspended in 0.1 M sodium carbonate buffer (pH 9.0) containing 1 mg/mL fluorescein isothiocyanate (FITC, cat. no. BS096-50 mg, Biosharp, China). After incubation at 37 °C for 1 h in the dark, labeled bacteria were washed three times with PBS and resuspended in DMEM at 5 × 10^7^ CFU/mL. RAW264.7 macrophages seeded on glass coverslips (5 × 10^5^ cells/well) were incubated with 100 μL of FITC-labeled bacterial suspension (MOI = 10) for 1 h at 37 °C with 5% CO_2_. After incubation, cells were washed three times with PBS, fixed with 4% paraformaldehyde for 30 min at room temperature in the dark, and washed again with PBS. Cell membranes were stained with DiD (cat. no. C1995S, Beyotime, China) for 15 min at room temperature in the dark, followed by PBS washes. Nuclei were stained with 4′,6-diamidino-2-phenylindole (DAPI, cat. no. BL105B, Biosharp, China) for 15 min. After final PBS washes, coverslips were mounted onto glass slides with antifade mounting medium. Images were acquired using a confocal laser scanning microscope. The entire experiment was performed with three independent biological replicates, each including triplicate wells per strain and time point.

### Cell adhesion and invasion assays

Adhesion and invasion capabilities of *S. agalactiae* strains were evaluated using TiB cells as previously described [23]. TiB cells were plated in 24-well plates at a density of 1 × 10^5^ cells per well and maintained in DMEM supplemented with 10% FBS. After reaching mid-exponential phase, *S. agalactiae* cultures were collected by centrifugation, subjected to three PBS washes, and then resuspended in DMEM at 1 × 10^7^ CFU/mL. Prior to infection, TiB cells were washed twice with PBS. Each well received 900 µL of DMEM with 10% FBS and 100 µL of the bacterial suspension, achieving a MOI of 10. After a centrifugation (800 × *g*, 10 min) to synchronize bacterium-cell contact, the plate was incubated at 28 °C for 1 h to allow for bacterial adhesion and invasion. Following incubation, the supernatant was aspirated, and monolayers were gently washed three times with PBS to remove non-adherent bacteria. For adhesion assay, the infected TiB cells were lysed by adding 1 mL of sterile ultrapure water per well, followed by 10-min incubation at room temperature and vigorous pipetting. The lysates were serially diluted ten-fold in PBS and plated on THB agar to quantify viable adherent bacteria. The adhesion rate was calculated as (CFU/mL recovered from adhered bacteria/CFU/mL in the initial inoculum) × 100%. For invasion assay, a separate set of infected TiB cells were cultured with 1 mL of DMEM containing penicillin (5 µg/mL) and gentamicin (100 µg/mL) for an additional 1 h at 28 °C to kill the extracellular *S. agalactiae*. Subsequently, the TiB cells were lysed as described above. To enumerate viable intracellular bacteria, the lysates underwent tenfold serial dilution in PBS before being plated onto THB agar. The percentage of invasion was calculated as (CFU/mL recovered after antibiotic treatment/CFU/mL in the initial inoculum) × 100%. Each experiment was performed with at least three independent biological replicates, with each strain tested in duplicate or triplicate wells per experiment.

### Cytotoxicity assay

Cytotoxicity against TiB cells was evaluated using a lactate dehydrogenase (LDH) release assay, following an established protocol [23]. After reaching mid-exponential phase, *S. agalactiae* cultures were collected by centrifugation, subjected to three PBS washes, and then resuspended in DMEM at a concentration of 1 × 10^7^ CFU/mL. To prepare for subsequent assays, TiB cells were plated in 24-well plates at a density of 1 × 10^5^ cells per well and maintained in complete DMEM (supplemented with 10% FBS). Prior to infection, TiB cell monolayers were rinsed twice with PBS. Each well was then inoculated with 0.1 mL of the bacterial suspension, achieving a multiplicity of infection (MOI) of 10. To ensure synchronous bacterial contact, the plate was briefly centrifuged (800 × *g*, 1 min) and subsequently transferred to a 37 °C incubator for a 4-h infection period. Following infection, cells and debris were pelleted by centrifugation at 400 × *g* for 5 min. From the resulting clarified supernatant, a 120 μL aliquot was carefully removed from each well and transferred to a fresh plate for the quantification of lactate dehydrogenase (LDH) activity. The LDH enzymatic activity was assessed using a commercial kit (cat. no. C0016, Beyotime, China), following the manufacturer’s instructions. Each experiment included three independent biological replicates.

### Survival analysis

Nile tilapia (*Oreochromis niloticus*, average weight 10 ± 2 g) were randomly divided into four groups (*n* = 30 per group): three challenge groups and one control group. Fish in the challenge groups were intraperitoneally (i.p.) injected with 1 × 10^6^ CFU/fish of the WT, Δ*corA*_*2*_, or CΔ*corA*_*2*_, suspended in 100 μL of sterile PBS. The control group received an equivalent volume of sterile PBS alone. Mortality was monitored twice daily for 14 d post-infection. For survival analysis, Kaplan–Meier curves were constructed and plotted with the aid of GraphPad Prism software. The protocol for all animal experiments was reviewed and approved by the Laboratory Animal Centre of Huazhong Agricultural University, China (ethical approval no. HZAUFI-2025-0123).

### Bacterial load and dissemination

To assess the colonization and dissemination capabilities of the *S. agalactiae* strains, a separate cohort of tilapia (*n* = 3 per group) was i.p. challenged with a dose of 1 × 10^4^ CFU/fish. At 72 h post-infection, fish were euthanized with MS-222 (500 mg/L), and tissues including the brain, liver, spleen, head kidney, and trunk kidney were aseptically collected. Each tissue sample was divided into two portions for subsequent bacterial load quantification, and the expression of immune factors. For bacterial load quantification, tissue samples were weighed and homogenized in sterile PBS to 0.5 mg/mL. The homogenates were serially diluted tenfold in PBS and plated onto THB agar plates. After incubation, the number of viable bacteria CFU was counted. The bacterial load was expressed as CFU per gram of tissue (Table 1).

**Table 1:** **Composition of the FMC medium (per liter, pH 7.0)**

Component	Concentration (mg/L)
Glucose	20 000 mg
Sodium acetate	6000 mg
(NH_4_)_2_SO_4_	600 mg
Adenine	35 mg
Guanine	27 mg
Uracil	30 mg
MgSO_4_	
NaCl	10 mg
FeSO_4_	10 mg
MnSo_4_	10 mg
KH_2_PO_4_	400 mg
K_2_HPO_4_	300 mg
Na_2_HPO_4_	3150 mg
NaH_2_PO_4_	2050 mg
Sodium citrate	225 mg
Riboflavin	0.4 mg
Biotin	0.01 mg
Folic acid	0.1 mg
Pantothenate	0.8 mg
*p*-Aminobenzoic acid	0.1 mg
Thiamine	0.4 mg
Nicotinamide	2 mg
Pyridoxamine	0.8 mg
Na_2_CO_3_	2014 mg
Glutamine	5 mg
Glutamic acid	300 mg
l-Aspartic acid	110 mg
l-Isoleucine	110 mg
l-Leucine	110 mg
l-Methionine	110 mg
l-Serine	110 mg
l-Phenylalanine	110 mg
l-Threonine	110 mg
l-Valine	100 mg
dl-Alanine	200 mg
l-Proline	200 mg
l-Arginine	200 mg
l-Cysteine	200 mg
l-Tryptophan	200 mg
Glycine	200 mg
l-Histidine	200 mg
l-Hydroxyproline	200 mg
l-Tyrosine	200 mg
l-Lysine	110 mg

### RNA extraction and quantitative real-time PCR (qRT-PCR)

Total RNA was extracted from head kidney tissues from the bacterial load assay cohort, using a total RNA extraction kit (cat. no. DP419, Tiangen Biotech, China). RNA quality, including concentration and purity, was assessed spectrophotometrically based on the A260/A280 ratio, with values between 1.8 and 2.1 indicating acceptable quality. Using the HiScript III cDNA Synthesis Kit (Vazyme, China), 1 μg of total RNA was converted into first-strand cDNA. The Bio-Rad CFX96 Real-Time PCR Detection System was used for qRT-PCR in conjunction with ChamQ SYBR qPCR Master Mix (Vazyme, China, cat. no. Q711-02). For data normalization, the *β-actin* gene served as the internal reference, and relative gene expression was quantified via the comparative 2^–ΔΔCt^ method. [27]. All qRT-PCR analyses included three biological replicates. Primer sequences are provided in Table 2.

**Table 2:** **The primers used in this study**

Primer name	Sequence (5' → 3′)	Function
*corA*_*2*_-up-F	CGGCCAGTGAATTCGAGCTCCCGACAGTGAGAACACCAAGTC	Left arm of *corA*_*2*_; amplifies the flank sequence located in coding region of *corA*_*2*_ upstream
*corA*_*2*_-up-R	AAAAATATTCGGTGCGTAAGCATAATCCATTTTTACCACTTCTAGC	
*corA*_*2*_-down-F	GCTAGAAGTGGTAAAAATGGATTATGCTTACGCACCGAATATTTTT	Right arm of *corA*_*2*_; amplifies the flank sequence located in coding region of *corA*_*2*_ downstream
*corA*_*2*_-down-R	GCATGCCTGCAGGTCGACGACACTGCTGGTATCCGTG	
*corA*_*2*_-IF	CCGTTCCGATGACCTTTTGC	A fragment for *corA*_*2*_ ORF; used to confirm the deletion of *corA*_*2*_. The WT will produce a fragment by PCR, but the mutant strain not
*corA*_*2*_-IR	CTGATGCGCTTCAATGAGAGC	
*corA*_*2*_-EF	TGAAGATATCGTGCGTGCGA	Genotypic verification of *corA*_*2*_ deletion by diagnostic PCR. Amplification yields a longer fragment in the WT and a shorter fragment in the mutant
*corA*_*2*_-ER	ATGGAGCACGAATGGCTGAA	
*CΔcorA*_2_-F	CGACGGCCAGTGAATTCTTATTTTCTTAAGTTAAAAACTCGTTTTAAAATTTGAG	Used for amplifying the *corA*_*2*_ fragment
*CΔcorA*_2_-R	GCCAAGCTTGCATGCAATGATAGTCGAACAAAAATTTGGAAAT	
GGGG-F	GGAGGAGGAGGAACCAATAAGCGCTTATTAGCC	Introducing a flexible 4-glycine linker between the α5 and α6 helices of *corA*_*2*_
GGGG-R	TCCTCCTCCTCCTGTTTGTTCTCTCAATTGACGGT	
D206K-F	ACAGTTATCAAAGGCTCTCATTGAAGCGCATC	Introducing the D206K point mutation in *corA*_*2*_
D206K-R	TTCAATGAGAGCCTTTGATAACTGTTCTTTTTC	
*IF-1β*-F	ATCAGTCCGTCGAAGACTCC	For qRT-PCR analysis
*IF-1β*-R	CAGAACACCAGCGTGGTTAG	
*IL-6*-F	ACAGAGGAGGCGGAGATG	
*IL-6*-R	GCAGTGCTTCGGGATAGAG	
*TNF-α*-F	GGTCATCTGGAGTGGAGGAA	
*TNF-α*-R	AGCCGTGGTCTGAGAAGCTA	
*β-actin*-F	GAGCGTGAGATTGTGCGTGAC	
*β-actin*-R	TCCATACCGAGGAATGAGGGC	

### Statistical analyses

Statistical analysis was conducted with GraphPad Prism (version 9.5). Unless otherwise noted in the figure legends, data from at least three independent biological replicates are expressed as mean ± standard deviation (SD). When comparing more than two groups, one-way analysis of variance (ANOVA) was applied. For comparisons between two groups, significance was assessed using an unpaired two-tailed Student’s *t*-test. A *p*-value of less than 0.05 was considered statistically significant (ns, not significant: *p* ≥ 0.05; *p < 0.05; **p < 0.01; ***p < 0.001; ****p < 0.0001).

## Results

### The role of CorA_2_ in magnesium transport and serum resistance

**Figure 1:**
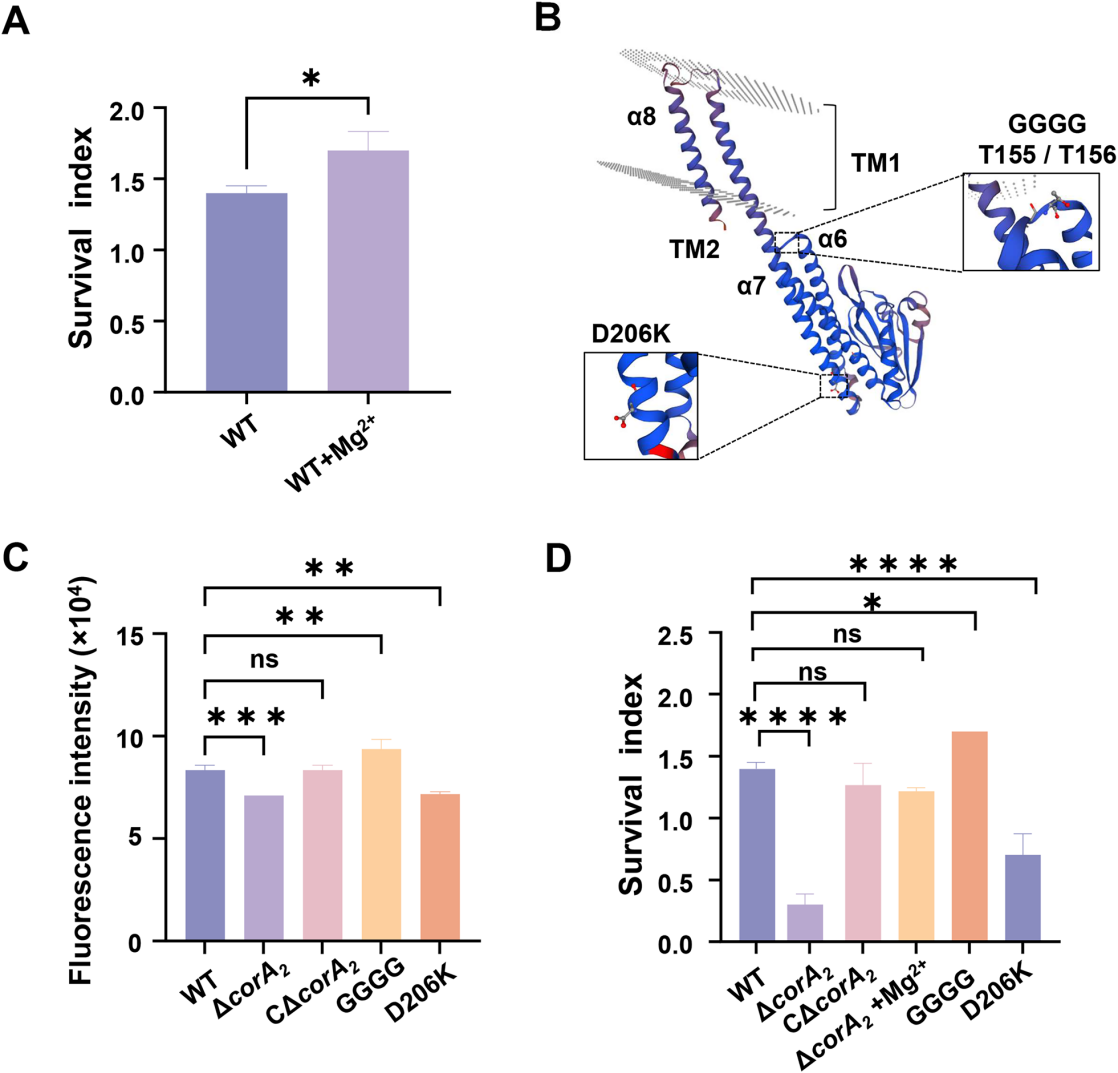
**CorA**_**2**_ **mediates magnesium uptake and is required for serum resistance in *S. agalactiae*. A** Survival index of the WT HN016 strain in tilapia serum with or without Mg^2+^ supplementation. Bacterial survival was quantified after 2 h of exposure. Data are presented as the survival index (mean ± SD; *n* = 3 independent experiments). **B** Schematic representation of the CorA_2_ transporter structure, highlighting the locations of the GGGG insertion and the D206K point mutation. **C** Intracellular Mg^2+^ levels in the WT, Δ*corA*_*2*_, CΔ*corA*_*2*_, GGGG, and D206K strains after 6 h of growth. Mg^2+^ concentration was measured using the Mag-Fluo-4 AM fluorescent probe. **D** Serum survival of the indicated strains after 2 h of exposure in tilapia serum. Data are presented as the survival index (mean ± SD; *n* = 3 independent experiments). Statistical analysis was performed using an unpaired Student’s *t*-test for panel **A** and one-way ANOVA for panels **C** and **D** (*p < 0.05, **p < 0.01, ***p < 0.001).

To determine whether extracellular Mg^2+^ enhances serum resistance, we compared the survival of the WT strain in tilapia serum with or without Mg^2+^ supplementation. The survival index increased by 1.2-fold in serum supplemented with Mg^2+^ compared with serum alone (1.40 ± 0.05 versus 1.70 ± 0.13; *p* < 0.05; Figure 1A), establishing the critical role of extracellular Mg^2+^ in resisting fish serum. To identify the transporter responsible for Mg^2+^ uptake, we constructed a *corA*_*2*_ deletion mutant (Δ*corA*_*2*_) (Additional file 4) and quantified intracellular Mg^2+^ levels. The Δ*corA*_*2*_ exhibited a 14.46% reduction in intracellular Mg^2+^ compared with the WT (*p* < 0.001; Figure 1C), a defect that was fully restored in the CΔ*corA*_*2*_. To further dissect the transport mechanism, we generated two structure-guided point mutants: a putative gain-of-function mutant (GGGG) and a loss-of-function mutant (D206K) (Figure 1B, Additional file 5). Consistent with their designed functions, the D206K mutant showed a 13.93% decrease in intracellular Mg^2+^, while the GGGG mutant accumulated 12.48% more Mg^2+^ than the WT (Figure 1C). The essential role of CorA_2_ in serum resistance was directly validated through serum challenge assays. The survival index of the Δ*corA*_*2*_ strain increased by approximately 4.1-fold upon Mg^2+^ supplementation (*p* < 0.001). Compared with the Mg^2+^-supplemented Δ*corA*_*2*_ strain, the survival index of the GGGG gain-of-function mutant was elevated by an additional ~ 1.4-fold, whereas that of the D206K loss-of-function mutant was reduced by ~ 57% (all *p* < 0.05; Figure 1D). Collectively, these data demonstrate that CorA_2_ is an essential Mg^2+^ transporter in *S. agalactiae*, and that its activity is essential for bacterial survival under host serum challenge.

### CorA_2_ is essential for growth under magnesium limitation and for resistance to stresses within a simulated lysosomal environment

**Figure 2:**
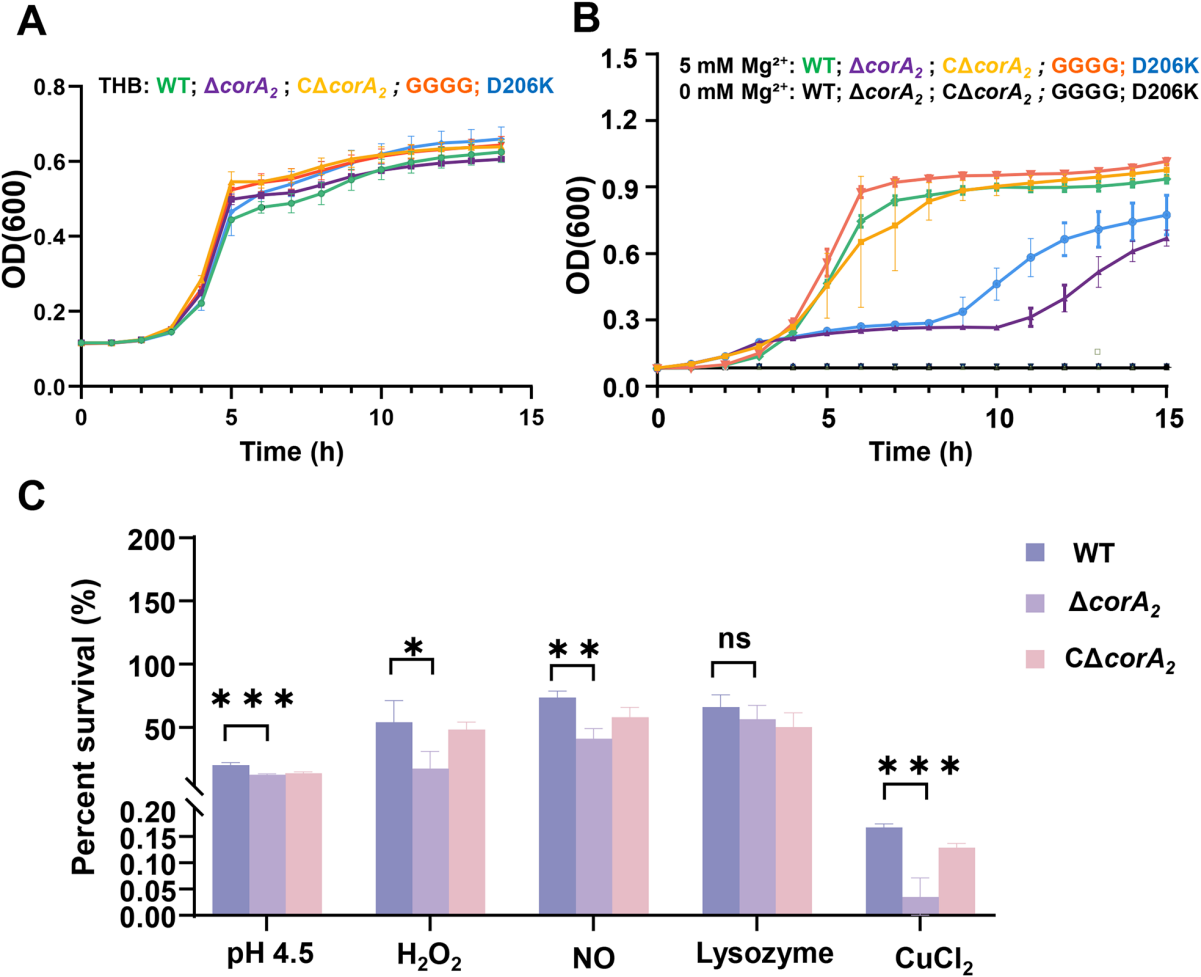
**CorA**_**2**_** is required for growth under magnesium limitation and for resistance to phagosome-mimicking stresses. A** Growth curves of WT, Δ*corA*_*2*_, CΔ*corA*_*2*_, GGGG, and D206K strains in THB. Growth was measured by measuring OD_600_ every 30 min for 15 h. Data are presented as mean OD600 ± standard deviation from three independent experiments. **B** Growth curves of the same strains in a chemically defined medium under without magnesium (0 mM MgSO_4_) or with magnesium (5 mM MgSO_4_) conditions. Growth was measured by measuring OD_600_ every 30 min for 15 h. Data are presented as mean OD_600_ ± standard deviation from three independent experiments. **C** Survival rates of the WT, Δ*corA*_*2*_, and CΔ*corA*_*2*_ strains following a 1-h exposure to various phagosome-mimicking stressors: oxidative stress (5 mM H_2_O_2_), acid nitrosative stress (NaNO_2_, pH 4.5), metal ion toxicity (CuCl_2_), enzymatic challenge (lysozyme), and acidic stress control (pH 4.5) Viable bacteria were quantified by plating serial dilutions on THB agar and enumerating CFU. Data are expressed as a percentage of survival relative to the untreated control. Data in all panels represent the mean ± standard deviation from three independent biological replicates. Statistical significance was determined by one-way ANOVA (*p < 0.05, **p < 0.01, ***p < 0.001).

To determine the physiological role of CorA_2_, the growth kinetics of the WT, Δ*corA*_*2*_, CΔ*corA*_*2*_, and the point mutants GGGG and D206K were monitored. In the nutrient rich THB medium, all strains exhibited comparable growth profiles (Figure 2A). However, under magnesium-limiting conditions, the Δ*corA*_*2*_ and D206K (loss-of-function) mutants exhibited severe growth defects, including a prolonged lag phase and a reduced growth rate. In contrast, the GGGG (gain-of-function) mutant showed a growth advantage, and the CΔ*corA*_*2*_ strain recovered a WT-like growth profile (Figure 2B). These results indicate that CorA_2_-mediated magnesium transport plays a crucial role in supporting the growth of *S. agalactiae* under magnesium restriction. We further assessed the role of CorA_2_ in stress adaptation by challenging the strains with distinct stressors. In the presence of lysozyme, all strains exhibited comparable survival rates. In contrast, under H_2_O_2_-induced oxidative stress, the survival rate of the Δ*corA*_*2*_ (17.34%) was significantly lower than that of the WT (53.91%, *p* < 0.05; Figure 2C). This susceptibility was rescued in the CΔ*corA*_*2*_ (48.35%; Figure 2C). Similarly, under Cu^2+^ stress, the Δ*corA*_*2*_ was highly vulnerable, with a survival rate of 0.04%, compared with 0.17% for the WT (*p* < 0.001), and complementation restored survival to 0.13% (Figure 2C). Under acidic stress (pH 4.5), the survival rate of Δ*corA*_*2*_ (12.30%) was significantly lower than that of the WT (20.13%, *p* < 0.001). Similarly, upon NO stress, the Δ*corA*_*2*_ mutant (40.81%) also showed a markedly reduced survival compared to the WT (73.45%, *p* < 0.01). Collectively, these findings demonstrate that CorA_2_ deficiency not only compromises growth under magnesium limitation but also markedly attenuates the ability of *S. agalactiae* to withstand oxidative, metal ion, acidic, and nitrosative stress.

### CorA_2_ is essential for evading phagocytosis, enabling intracellular survival, and executing host cell damage

**Figure 3:**
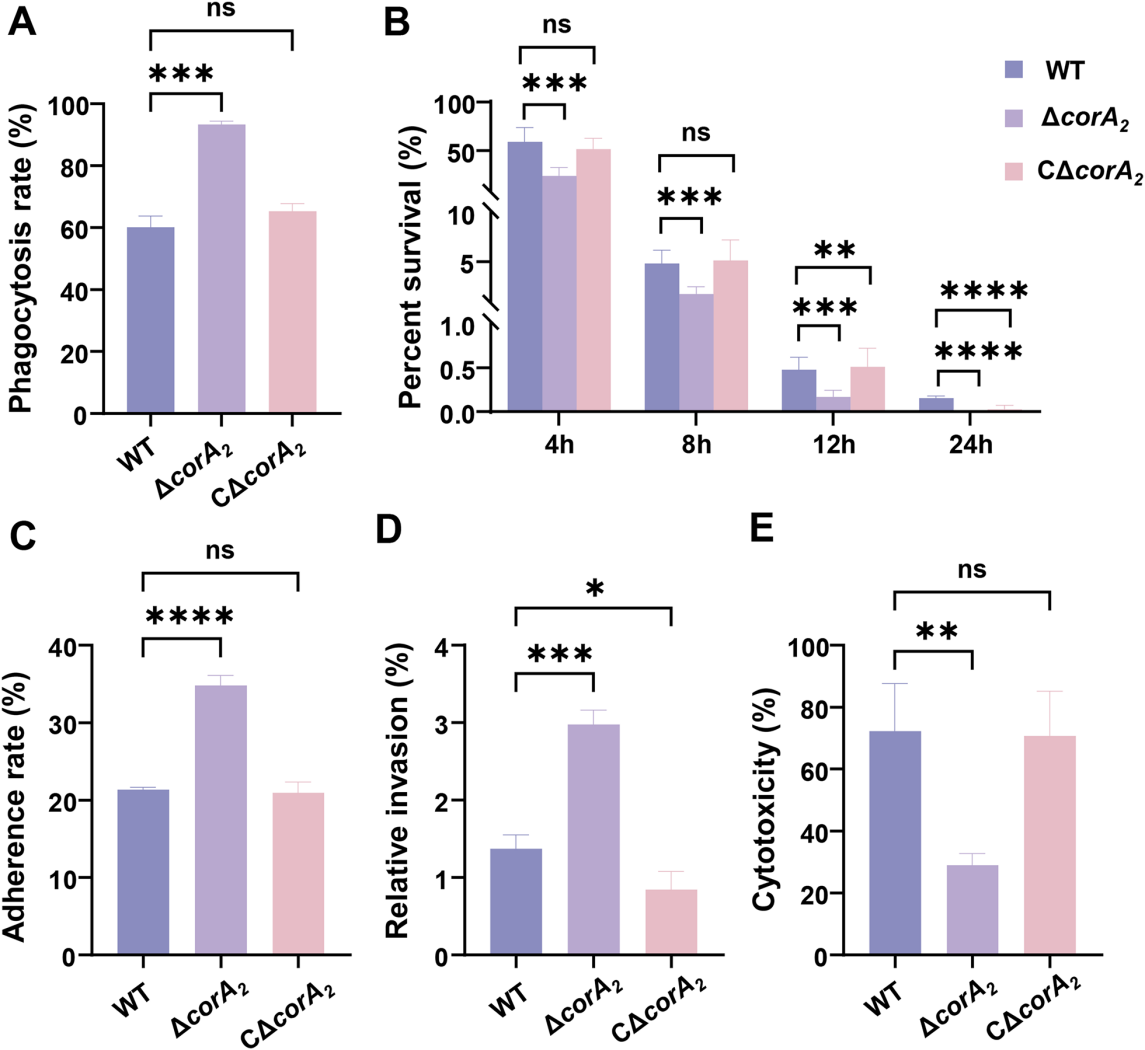
**CorA**_**2**_** deficiency impairs bacterial interactions with host cells by reducing intracellular survival and cytotoxicity despite enhancing adhesion and invasion. A** Phagocytosis rates of the WT, Δ*corA*_*2*_, and CΔ*corA*_*2*_ strains by murine RAW 264.7 macrophages. The phagocytosis rates were determined after a 1-h infection. **B** Intracellular survival of the WT, Δ*corA*_*2*_, and CΔ*corA*_*2*_ strains within RAW 264.7 macrophages over 24 h. Data are expressed as the percentage of bacteria that survived relative to the initial number of internalized bacteria (0-h time point). **C** Adhesion rates of the WT, Δ*corA*_*2*_, and CΔ*corA*_*2*_ strains to tilapia TIB cells after 1 h of infection. **D** Invasion rates of the WT, Δ*corA*_*2*_, and CΔ*corA*_*2*_ strains into TIB cells. The percentage of invasive bacteria was determined after a 1-h infection followed by an antibiotic protection period to kill extracellular bacteria. **E** Cytotoxicity of the WT, Δ*corA*_*2*_, and CΔ*corA*_*2*_ strains towards TIB cells. The cytotoxicity was measured by lactate dehydrogenase (LDH) release after 4 h of infection. Data are expressed as the percentage of total cellular LDH release relative to the maximum LDH release from lysed control cells. For all panels, data are presented as the mean ± standard deviation from three independent biological replicates. Statistical significance was determined by one-way ANOVA. (*p < 0.05, **p < 0.01, ***p < 0.001, ****p < 0.0001).

To define the role of CorA_2_ in host–pathogen interactions, we assessed its impact on immune evasion using murine macrophages and on cytotoxicity using TiB cells. The Δ*corA*_*2*_ mutant exhibited a profound defect in evading phagocytic clearance, showing a significantly higher uptake rate than the WT strain (93.3% versus 60.2%; *p* < 0.001; Figure 3A). This difference was corroborated by confocal imaging, which showed sparse intracellular WT bacteria but dense clusters of internalized Δ*corA*_*2*_ bacteria within macrophages (Additional file 6). Once internalized, the mutant’s intracellular survival was severely compromised, with its viability plummeting to 23.9% at 4 h compared with 59.2% for the WT (*p* < 0.001). By 24 h, the mutant was completely eradicated, whereas a small population of WT persisted (0.15%; *p* < 0.001; Figure 3B). We further examined the interaction between the bacteria and TiB cells. Interestingly, the Δ*corA*_*2*_ exhibited significantly enhanced adhesion (34.8% vs. 21.4% for WT; *p* < 0.05; Figure 3C) and invasion (3.0% versus 1.4% for WT; *p* < 0.001; Figure 3D). Despite this heightened adhesion and invasion, the Δ*corA*_*2*_ was severely compromised in its capacity to damage the host. The cytotoxicity induced by the Δ*corA*_*2*_ (29.0%) was less than half of that caused by the WT strain (72.3%) (Figure 3E). All these defects in Δ*corA*_*2*_ were fully rescued to WT levels in the CΔ*corA*_*2*_ (Figure 3). Collectively, these data demonstrate that CorA_2_ is indispensable for resisting phagocytosis, enabling intracellular survival, and ultimately executing host cell damage, underscoring its multifaceted role in virulence.

### CorA_2_ is essential for the virulence of ***S. agalactiae***

**Figure 4:**
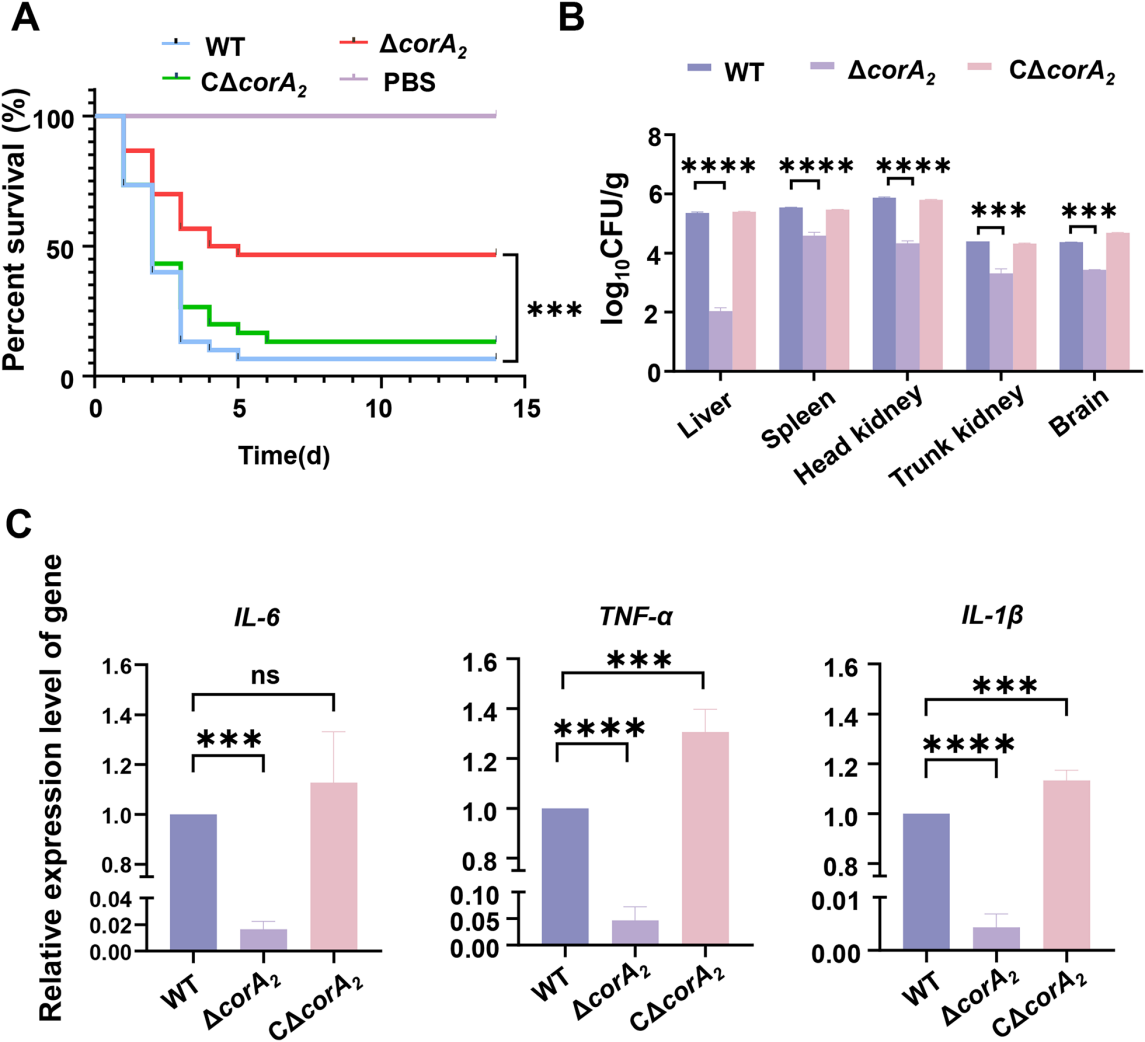
***corA***_***2***_** is essential for the virulence of *S. agalactiae* in a tilapia infection model. A** Survival analysis of tilapia following intraperitoneal challenge with the WT, Δ*corA*_*2*_, or CΔ*corA*_*2*_ strain (1 × 10^6^ CFU/fish). Mortality was monitored for 14 days post-infection (*n* = 30 fish per group). **B** Bacterial colonization in systemic tissues at 72 h post-infection. Tilapia were challenged with a lower dose (1 × 10^4^ CFU/fish), and bacterial loads in the indicated organs were quantified by plate counting. Data are presented as log_10_ CFU per gram of tissue (mean ± SD; *n* = 3 fish per group). **C** Expression levels of pro-inflammatory cytokines in the head kidney of infected tilapia. The relative mRNA expression of *IL*-*6*, *TNF*-*α*, and *IL*-*1β* was measured by qRT-PCR at 72 h post-infection. Data are normalized to the *β*-actin reference gene and presented as mean ± SD (*n* = 3). For all panels, statistical significance was determined by the log-rank test (**A**) or one-way ANOVA (**B**, **C**) (*p < 0.05, **p < 0.01, ***p < 0.001).

To further assess the role of CorA_2_ in pathogenesis, the survival rate, bacterial colonization, and host immune responses were evaluated after the tilapia were infected with WT, Δ*corA*_*2*_, and CΔ*corA*_*2*_. The survival rate of the fish infected with WT and CΔ*corA*_*2*_ were only 6.7% and 13.3%, respectively, (Figure 4A). In stark contrast, infection with Δ*corA*_*2*_ resulted in a significantly higher survival rate of 46.7% (*p* < 0.001; Figure 4A). The attenuated virulence was directly linked to a profound defect in systemic dissemination. At 72 h post-infection, the bacterial loads of the Δ*corA*_*2*_ mutant were drastically reduced across all examined tissues compared with the WT (Figure 4B). The most pronounced reduction was observed in the liver, where the mutant’s load was approximately 2100-fold lower (*p* < 0.001). Significant reductions were also evident in the head kidney (~34-fold), spleen (~8.8-fold), trunk kidney (~11.5-fold), and brain (~8.6-fold) (all *p* < 0.001). Complementation with CΔ*corA*_*2*_ restored bacterial loads to levels comparable to the WT (Figure 4B). Consistent with the reduced bacterial burden, the hyper-inflammatory response typically triggered by infection was also mitigated. The expression of key pro-inflammatory cytokines (*IL*−1*β*, *TNF-α*, *IL*−6) in the head kidney was significantly downregulated in the Δ*corA*_*2*_ infected group compared with the WT and CΔ*corA*_2 _infected groups (Figure 4C). In summary, CorA_2_ deficiency severely cripples the *S. agalactiae* ability to establish a lethal infection, thereby confirming that CorA_2_ is essential for the virulence of *S. agalactiae *in vivo.

## Discussion

Mg^2+^ homeostasis is critical for cell physiology and is tightly regulated by specialized transporters, including the CorA, MgtA/B, MgtE, and Nramp families [28]. While these systems are well-characterized in some pathogens, their roles in *S. agalactiae* remain largely unexplored. Our functional analysis establishes CorA_2_ as an essential Mg^2+^ transporter in *S. agalactiae*. The severe intracellular Mg^2+^ deficiency observed in the Δ*corA*_*2*_ and D206K mutants, coupled with the elevated Mg^2+^ levels in the GGGG insertion mutant, establishes CorA_2_’s essential and regulated role in Mg^2+^ acquisition. This functional characterization is supported by structure-guided mutagenesis: the D206K mutation disrupts a key Mg^2+^-sensing site through charge substitution, effectively locking the channel in a closed conformation as previously demonstrated in TmCorA [19]. Conversely, the GGGG insertion within the α5–α6 loop increases conformational flexibility, promoting channel opening and constitutive Mg^2+^ influx, analogous to the gain-of-function Gly_4_-insertion mutants reported in TmCorA [19]. The observation that the Δ*corA*_*2*_ mutant grows identically to the WT under nutrient-rich conditions suggests functional redundancy. This compensatory mechanism may explain the viability of the *corA*_*2*_ mutant. It is worth noting that, although the survival index of the Mg^2+^ supplemented Δ*corA*_*2*_ mutant showed no statistically significant difference compared to WT, it remained numerically slightly lower. This observation suggests that, while CorA_2_-mediated magnesium transport is its primary function, the existence of additional moonlighting functions cannot be entirely excluded.

CorA family proteins serve as crucial transport systems for bacterial magnesium acquisition under low-magnesium conditions [14]. In this study, severe growth defects were observed for both Δ*corA*_*2*_ and D206K mutants under magnesium restriction, while no phenotypic difference was detected in nutrient-rich THB medium compared with the WT. These findings indicate that CorA_2_ is essential for Mg^2+^ uptake in low-magnesium environments. Consistent with observations in the pathogenic fungus role of CorA-family transporters in magnesium acquisition and bacterial fitness. In addition, compared with *Helicobacter pylori*, which relies solely on a single CorA system and whose mutants require supplementation with as high as 20 mM Mg^2+^ to restore growth [14], our study suggests that *S. agalactiae* may utilize multiple CorA homologs to achieve functional specialization and coordination across varying magnesium concentrations. This adaptive mechanism likely enhances the bacterium’s ability to survive and thrive within the complex and dynamic magnesium landscape of the host environment. The concurrent hypersensitivity to oxidative, metal ion, acidic, nitrosative stress and phagocytic clearance definitively establishes CorA_2_ as a central virulence factor. It is also worth noting that all strains exhibited comparable survival under lysozyme stress. Although Mg^2+^ can interact with lipoteichoic acid (LTA) and LTA has been shown to influence lysozyme susceptibility [29], this interaction is weak (*K*_d_ ≈ 15 mM [30]). Thus, reduced intracellular Mg^2+^ in Δ*corA*_*2*_ is insufficient to alter LTA structure or lysozyme susceptibility. We propose that intracellular magnesium shortage impairs ribosomal stability and function, thereby compromising the synthesis of proteins required for resisting phagosomal stresses and inducing host cell damage, consistent with the significantly reduced cytotoxicity of the Δ*corA*_*2*_ mutant. This hypothesis is supported by multiple lines of evidence. First, magnesium has long been recognized as a critical structural component of the ribosome, stabilizing the association between ribosomal subunits and facilitating proper protein synthesis [31]. Second, studies in *Escherichia coli* have demonstrated that magnesium deprivation leads to rapid ribosomal subunit dissociation and a consequent cessation of protein synthesis [32]. Third, a recent study showed that magnesium influx prevents membrane hyperpolarization and cell death following ribosome-targeting antibiotic treatment, thereby enhancing bacterial survival under ribosomal stress [33]. The Δ*corA*_*2*_ mutant exhibited enhanced adhesion to and invasion of tilapia brain cells. This phenotype mirrors observations in the unencapsulated mutant of *S. agalactiae* [34]. We hypothesize that CorA_2_ deficiency alters the bacterial surface, potentially by reducing capsular polysaccharide (CPS) synthesis. This hypothesis is supported by two key observations. One is that CPS impedes adhesion through steric and electrostatic hindrance [34]. Another one is that repressed CPS production increases adhesion and invasion in *S. agalactiae* [35]. Directly supporting this mechanism, our transmission electron microscopy analysis demonstrated that the Δ*corA*_*2*_ mutant possesses a thinner capsule than the complemented strain, a defect that was rescued by Mg^2+^ supplementation (Additional file 7). Furthermore, capsule production depends on the Mg^2+^-dependent enzyme CpsA [36], and the intracellular magnesium shortage in the ΔcorA2 likely curtails CPS assembly (Additional file 8). Consequently, the reduced capsule coverage unmasks underlying adhesins, leading to the observed enhancement in adhesion and invasion, a mechanism previously documented in *S. agalactiae* [35].

Our in vivo tilapia challenge model confirmed the severe attenuation of Δ*corA*_*2*_ mutant virulence. This finding aligns with observations in the plant pathogen *Pectobacterium carotovorum*, where CorA deletion similarly impaired pathogenicity and survival [13]. Similarly, in *Salmonella enterica* serovar Typhimurium, CorA is required for full virulence in mice, and *corA* mutants exhibit defects in epithelial cell invasion and intracellular replication [18]. These cross-kingdom conserved observations suggest that CorA family transporters play a broad and conserved role in bacterial pathogenesis. The critical importance of magnesium acquisition during infection is further underscored by the host’s SLC11A1 protein, which restricts intracellular magnesium to control *S.* Typhimurium growth [11]. Additionally, Δ*corA*_*2*_ exhibited significantly reduced bacterial loads in all examined tissues, with the most pronounced reduction observed in the liver (~ 2100-fold). Kupffer cells in the liver express the CRIg receptor, which directly binds LTA on the surface of Gram-positive bacteria, mediating rapid capture and clearance [37]. Although this clearance mechanism operates on both WT and mutant strains, the WT likely resists it through intact capsule or other virulence factors, whereas Δ*corA*_*2*_, owing to impaired magnesium uptake and increased susceptibility to phagocytic killing, loses this resistance. Other tissues lack this highly efficient direct recognition receptor, explaining the relatively modest reductions observed in these organs. These findings suggest that CorA_2_-mediated magnesium transport may play a critical role in resistance hepatic-specific immune clearance. Our study showed that pro-inflammatory cytokine levels were significantly lower in Δ*corA*_*2*_-infected fish compared with WT-infected fish, consistent with the markedly reduced bacterial loads in all tissues. This indicates that the attenuated inflammatory response reflects lower bacterial burden rather than active immunosuppression, as bacterial load is known to drive inflammation [38, 39]. While excessive inflammation causes tissue damage, the rapid clearance of Δ*corA*_*2*_ without overt inflammation is beneficial in acute infection. Whether this attenuated response is sufficient for long-term protective immunity remains to be determined. This study demonstrates that CorA_2_ serves as a key system for counteracting nutritional immunity in *S. agalactiae*. The Δ*corA*_*2*_ mutant fails to effectively colonize and disseminate in the magnesium-depleted host environment, ultimately leading to its clearance. The attenuation mechanism of Δ*corA*_*2*_ is based on nutritional limitation of magnesium. This feature is advantageous for vaccine immunogenicity, as limited in vivo replication can stimulate durable immune responses. Studies have shown that auxotrophic live attenuated vaccines utilizing this mechanism confer effective protection against *S. agalactiae* in tilapia models [40]. Consistent with findings from other nutrient metabolism-related gene deletion mutants [41], nutritional limitation-based attenuation strategies exhibit promising potential for vaccine development. Given this profound and specific attenuation phenotype and its capacity for sustained immunity, the Δ*corA*_*2*_ strain represents a promising candidate for developing a novel live attenuated vaccine against piscine streptococcosis. Nevertheless, further optimization is warranted. The Δ*corA*_*2*_ mutant retains partial magnesium uptake capacity, suggesting potential functional compensation by its homolog CorA. Therefore, construction of a *corA*/*corA*_*2*_ double deletion mutant would help further consolidate the attenuated phenotype. Additionally, the safety of Δ*corA*_*2*_ in immunocompromised hosts has not yet been evaluated and requires future investigation.

This study is not without limitations. First, the lack of a CorA_2_-specific antibody precluded direct Western blot validation of mutant protein stability and localization. Future investigations employing epitope-tagged CorA_2_ constructs will enable definitive validation of protein expression and facilitate subcellular localization studies. Second, the function of the CorA homolog remains to be investigated, as Δ*corA*_*2*_ retained partial magnesium uptake. Third, while Δ*corA*_*2*_ shows attenuated virulence, its vaccine potential requires further evaluation of safety, immunogenicity, and protective efficacy. Future studies addressing these limitations will provide a more complete understanding of CorA_2_ function.

In summary, while Mg^2+^ homeostasis is a fundamental aspect of cellular physiology, its specific regulation in *S. agalactiae* has remained poorly defined. This study definitively establishes the CorA_2_ transporter as a master regulator of Mg^2+^ homeostasis in this significant pathogen. The Δ*corA*_*2*_ mutant failure to survive within macrophages, and its consequent attenuation in the tilapia model, collectively demonstrate that CorA_2_-mediated magnesium acquisition is a cornerstone of virulence. By elucidating this critical vulnerability, our work not only advances the understanding of *S. agalactiae* pathogenesis but also provides a rationally designed, attenuated strain as a promising candidate for the development of a novel live vaccine against piscine streptococcosis.

## Supplementary Information

**Additional file 1: Sequence and structural comparison of SAHN016 proteins with the Mg**^**2+**^ **transporter TmCorA.** Multiple sequence alignment. Amino acid sequences of the known Mg^2+^ transporter TmCorA and two SAHN016 proteinswere aligned using the MUSCLE algorithm. Identical residues are highlighted. Structural superposition and RMSD analysis. Predicted structural models of the two SAHN016 proteins were superimposed onto the template protein TmCorA. The overall root-mean-square deviationvalues for Cα atoms are indicated. SAHN016_04670 shows the smallest RMSD to TmCorA, while SAHN016_02720 exhibits the largest structural divergence.

**Additional file 2: Validation of Mag-Fluo-4 AM dye loading efficiency in different strains.** Bacterial cells of WT, Δ*corA*2, CΔ*corA*2, GGGG, and D206K strains at logarithmic phase were stained with Mag-Fluo-4 AM for 30 min, followed by sonication for 15 min. Exogenous Mg^2+^ was added at final concentrations of 5, 10, and 15 mM to saturate the dye, and fluorescence intensity was measured. Statistical analysis was performed using one-way ANOVA. Data are presented as mean ± SD from three independent biological replicates.

**Additional file 3: Validation of antibiotic efficacy against** ***S. agalactiae*** **strains**. WT, Δ*corA*2, and CΔ*corA*2treated with or without penicillinand gentamicinat 37 °C with 5% CO_2_ for 1 h, after which 100 µL was plated onto antibiotic-free THB agar. After 24 h of incubation at 37 °C, colony growth was assessed.

**Additional file 4: Validation by PCR for the** ***corA2*** **deletion strain**. Lane M: DL2000 DNA Marker. Lanes 1 and 5: amplification with internal primers using WT and Δ*corA*2 genomic DNA as template, respectively. Lanes 2 and 6: amplification with external primers using WT and Δ*corA*2 genomic DNA as template, respectively. Lanes 3 and 4: negative controls.

**Additional file 5: Sequencing analysis for point mutation validation.**Sequencing identification of the D206K point mutation. DNA fragments around the target site were amplified by PCR and then subjected to sequencing analysis. The arrow indicates the position where the lysinecodon replaces the aspartic acidcodon. The chromatogram shows a single base substitutionat this site.Sequencing identification of the GGGG insertion mutation. DNA fragments around the target site were amplified by PCR and then subjected to sequencing analysis. The arrow indicates the insertion site of the four glycinecodons.

**Additional file 6: Confocal microscopy analysis of macrophage phagocytosis of WT and** ***ΔcorA2*** **strains**. Representative confocal images of RAW264.7 macrophages infected with FITC-labeled WT or Δ*corA*2 bacteriaat an MOI of 10 for 1 h. Cell membranes were stained with DiDand nuclei with DAPI. Scale bar, 5 μm.

**Additional file 7: CorA2 affects capsular content.**Transmission electron microscopy images ofCΔ*corA*2,Δ*corA*2, andΔ*corA*2 supplemented with exogenous Mg^2+^. Scale bar: 500 nm

**Additional file 8: Expression analysis of the capsule synthesis key gene** ***Acps***. Transcriptional levels of cpsA in WT, Δ*corA*2, CΔ*corA*2, GGGG, and D206K strains were determined by qRT-PCR. The 16S rRNA gene was used as an internal reference gene. All data are presented as mean ± SD from three independent biological replicates. Statistical significance was determined by one-way ANOVA.

## Data Availability

The datasets supporting the findings of this study are available within the main article and its supplementary materials.
