# Supplementary figures and images for: CorA2-mediated magnesium transport is essential for stress adaptation and virulence of Streptococcus agalactiae
Source: Vet Res. 2026 Apr 21;57:94. doi: 10.1186/s13567-026-01757-3 (PMC13214224; doi:10.1186/s13567-026-01757-3)

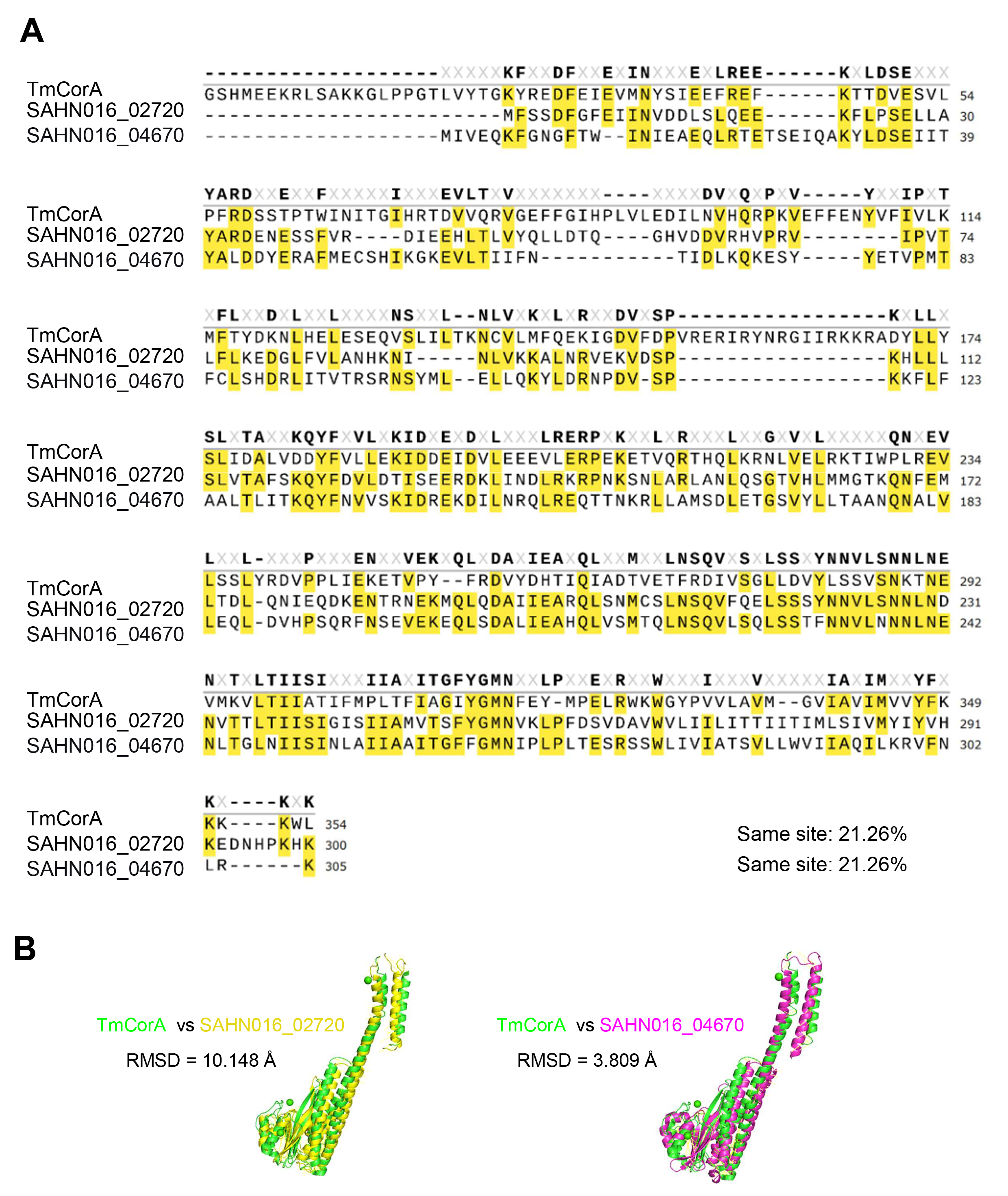

Supplement: Supplementary file 1 — Additional file 1: Sequence and structural comparison of SAHN016 proteins with the Mg2+ transporter TmCorA. Multiple sequence alignment. Amino acid sequences of the known Mg2+ transporter TmCorA and two SAHN016 proteinswere aligned using the MUSCLE algorithm. Identical residues are highlighted. Structural superposition and RMSD analysis. Predicted structural models of the two SAHN016 proteins were superimposed onto the template protein TmCorA. The overall root-mean-square deviationvalues for Cα atoms are indicated. SAHN016_04670 shows the smallest RMSD to TmCorA, while SAHN016_02720 exhibits the largest structural divergence. [file 13567_2026_1757_MOESM1_ESM.tif]

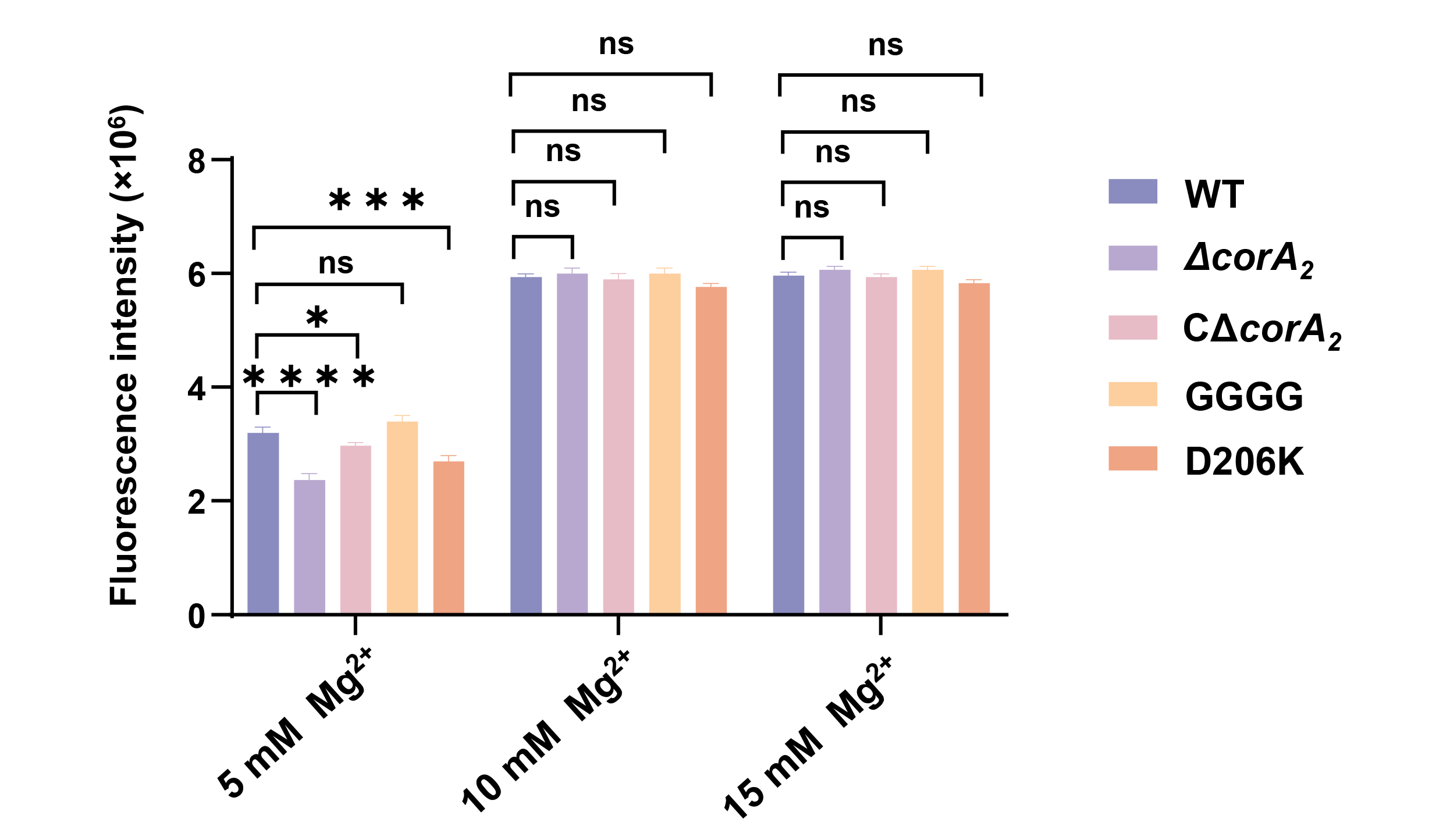

Supplement: Supplementary file 2 — Additional file 2: Validation of Mag-Fluo-4 AM dye loading efficiency in different strains. Bacterial cells of WT, ΔcorA2, CΔcorA2, GGGG, and D206K strains at logarithmic phase were stained with Mag-Fluo-4 AM for 30 min, followed by sonication for 15 min. Exogenous Mg2+ was added at final concentrations of 5, 10, and 15 mM to saturate the dye, and fluorescence intensity was measured. Statistical analysis was performed using one-way ANOVA. Data are presented as mean ± SD from three independent biological replicates. [file 13567_2026_1757_MOESM2_ESM.tif]

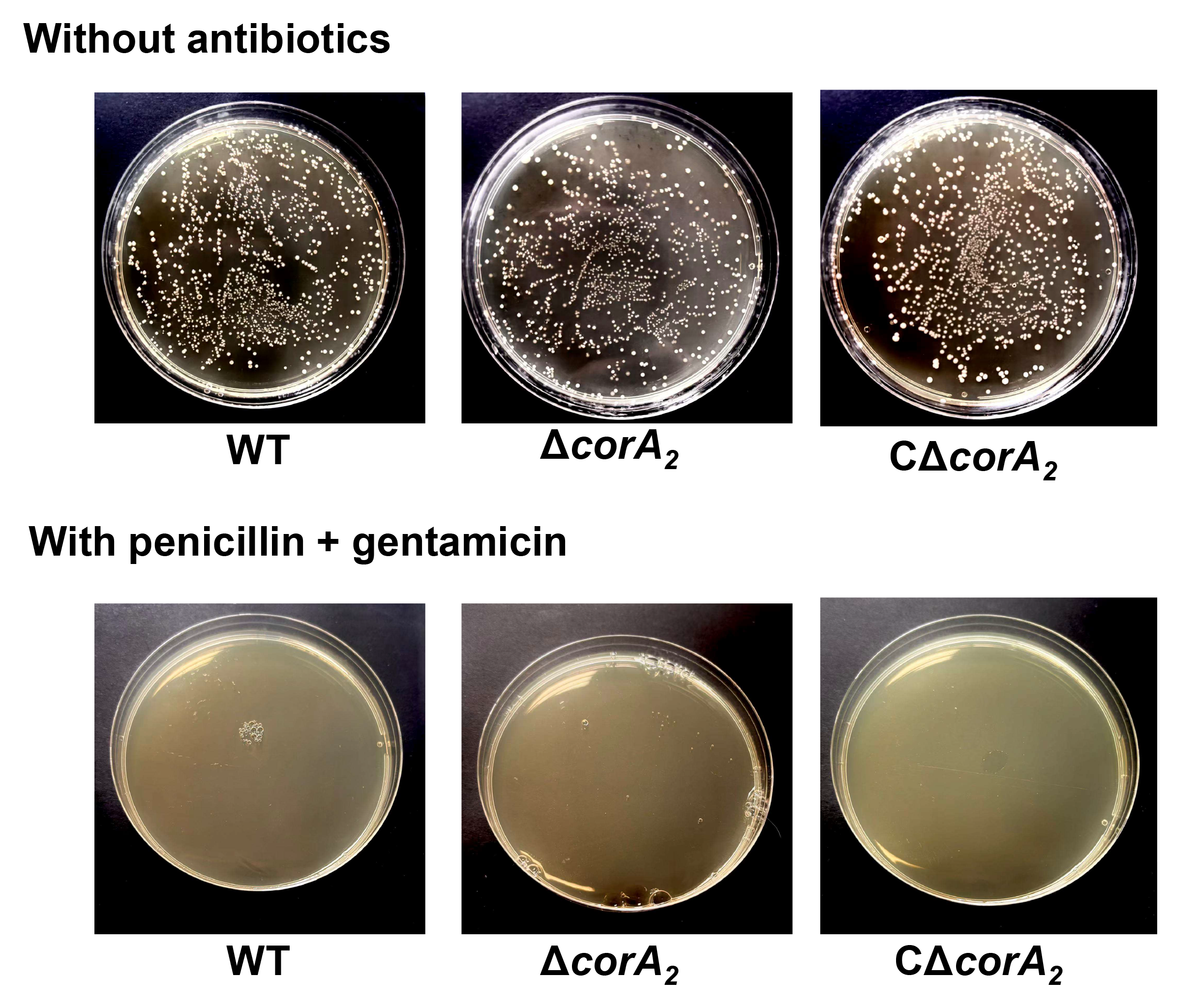

Supplement: Supplementary file 3 — Additional file 3: Validation of antibiotic efficacy against S. agalactiae strains. WT, ΔcorA2, and CΔcorA2treated with or without penicillinand gentamicinat 37 °C with 5% CO2 for 1 h, after which 100 µL was plated onto antibiotic-free THB agar. After 24 h of incubation at 37 °C, colony growth was assessed. [file 13567_2026_1757_MOESM3_ESM.tif]

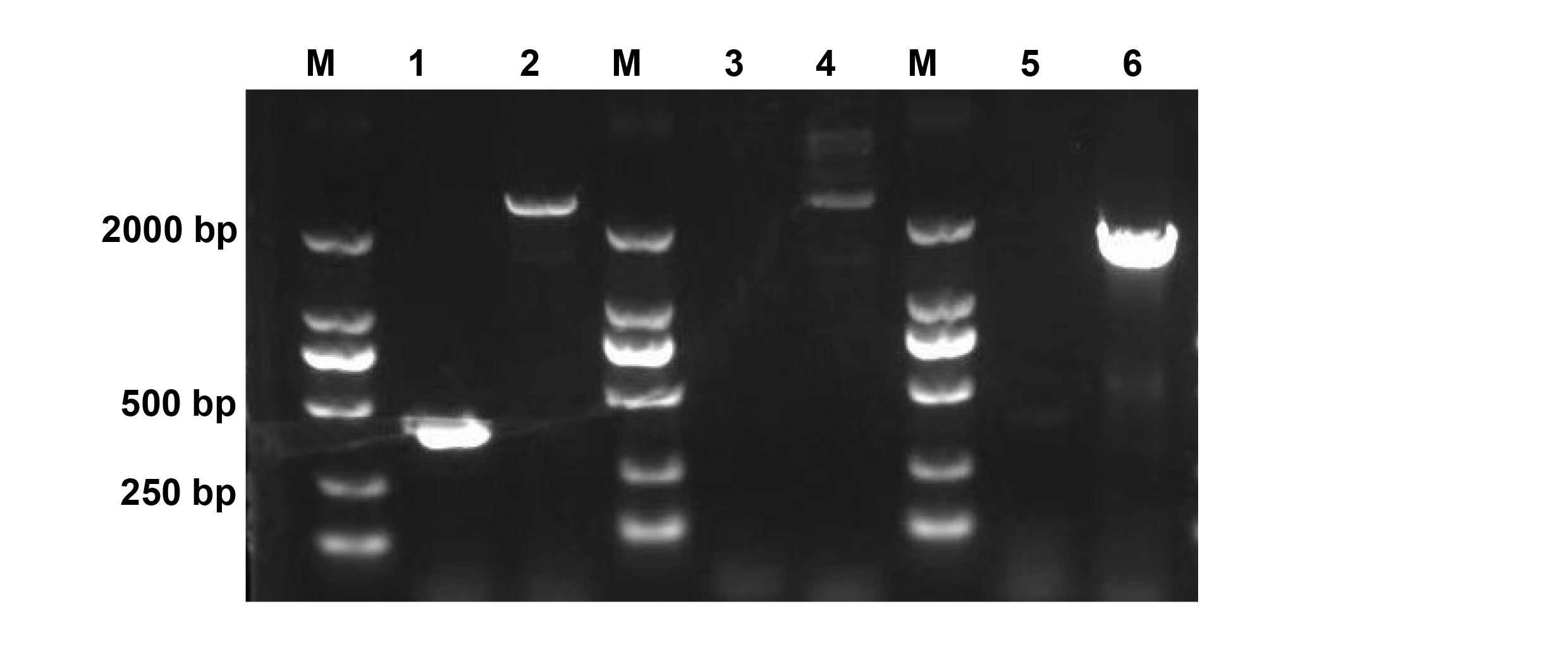

Supplement: Supplementary file 4 — Additional file 4: Validation by PCR for the corA2 deletion strain. Lane M: DL2000 DNA Marker. Lanes 1 and 5: amplification with internal primers using WT and ΔcorA2 genomic DNA as template, respectively. Lanes 2 and 6: amplification with external primers using WT and ΔcorA2 genomic DNA as template, respectively. Lanes 3 and 4: negative controls. [file 13567_2026_1757_MOESM4_ESM.tif]

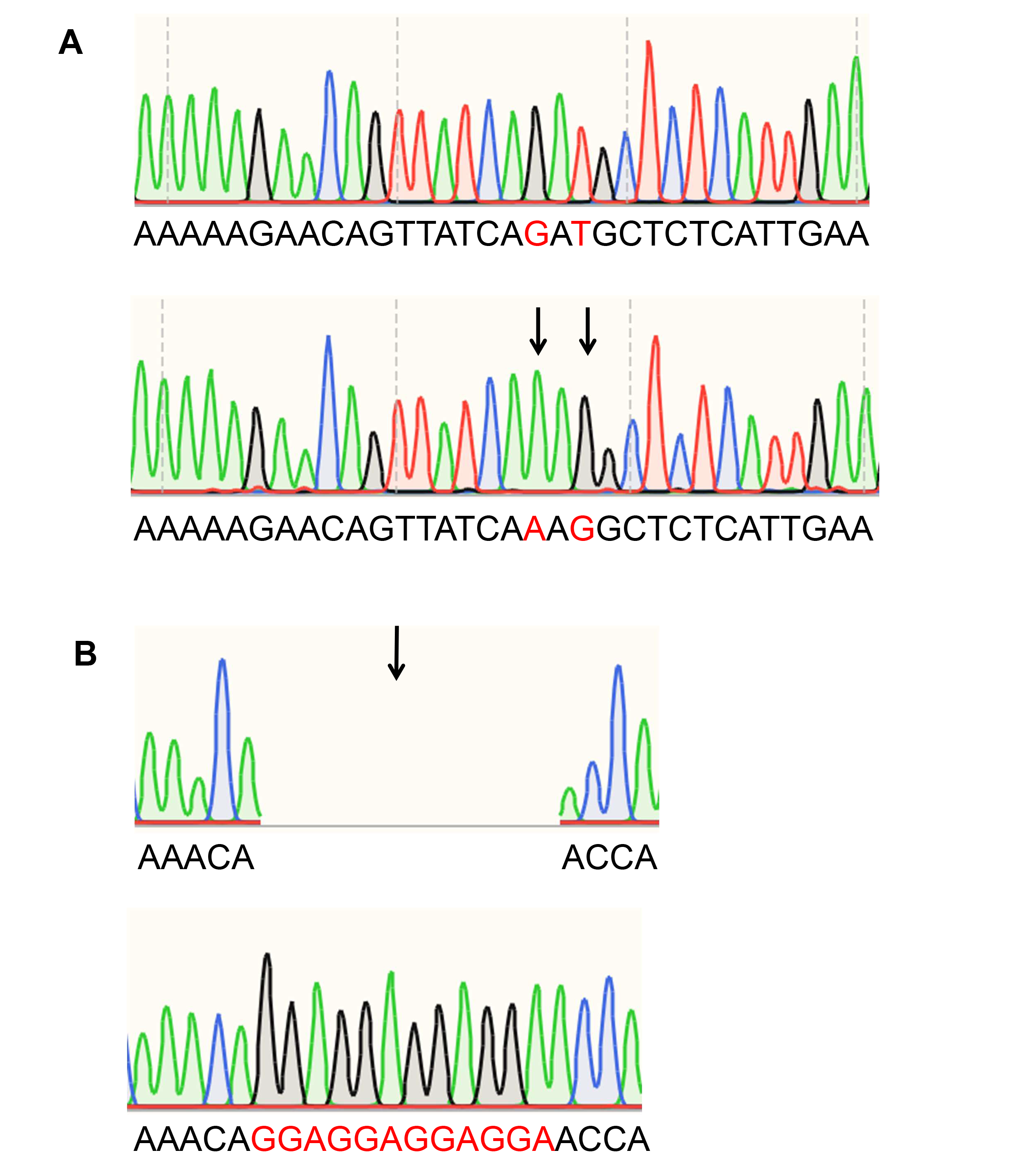

Supplement: Supplementary file 5 — Additional file 5: Sequencing analysis for point mutation validation.Sequencing identification of the D206K point mutation. DNA fragments around the target site were amplified by PCR and then subjected to sequencing analysis. The arrow indicates the position where the lysinecodon replaces the aspartic acidcodon. The chromatogram shows a single base substitutionat this site.Sequencing identification of the GGGG insertion mutation. DNA fragments around the target site were amplified by PCR and then subjected to sequencing analysis. The arrow indicates the insertion site of the four glycinecodons. [file 13567_2026_1757_MOESM5_ESM.tif]

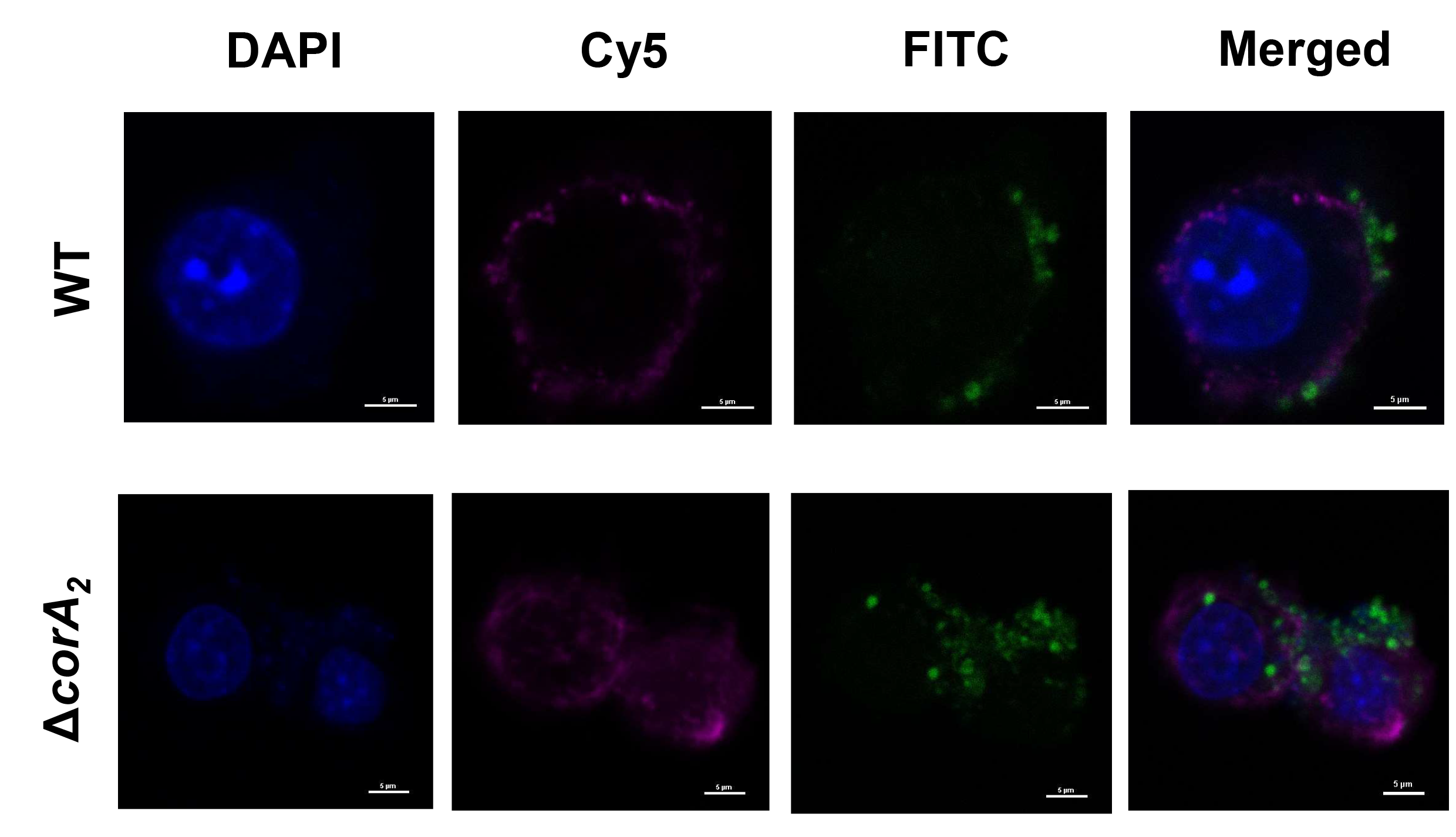

Supplement: Supplementary file 6 — Additional file 6: Confocal microscopy analysis of macrophage phagocytosis of WT and ΔcorA2 strains. Representative confocal images of RAW264.7 macrophages infected with FITC-labeled WT or ΔcorA2 bacteriaat an MOI of 10 for 1 h. Cell membranes were stained with DiDand nuclei with DAPI. Scale bar, 5 μm. [file 13567_2026_1757_MOESM6_ESM.tif]

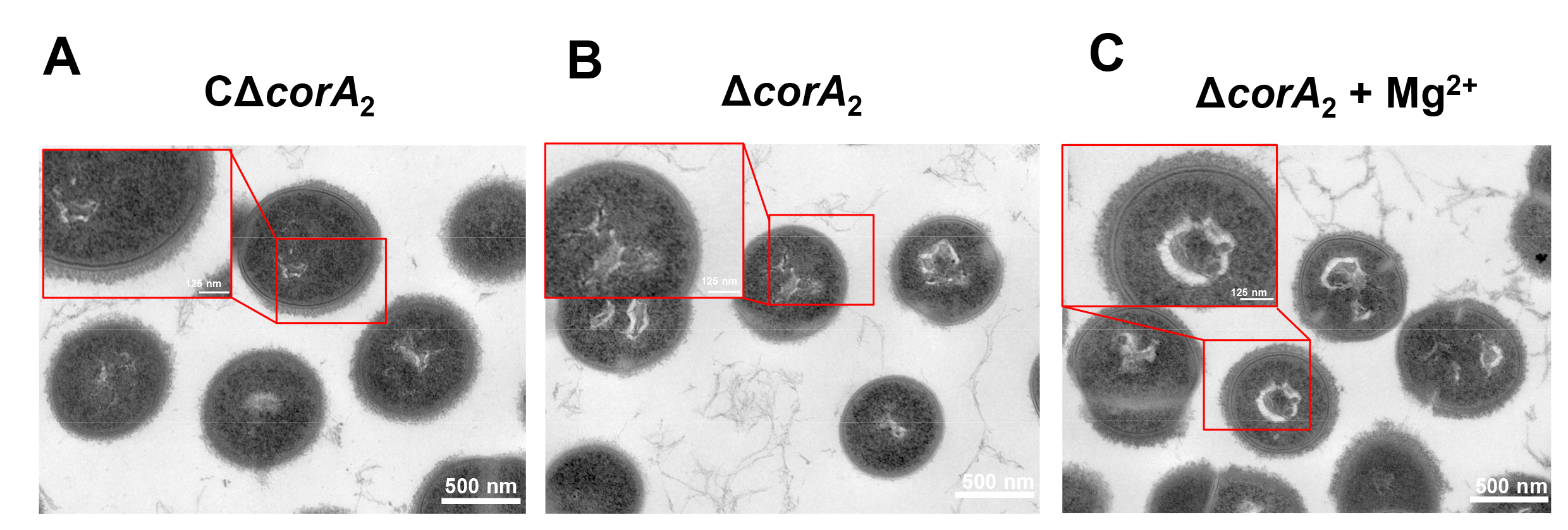

Supplement: Supplementary file 7 — Additional file 7: CorA2 affects capsular content.Transmission electron microscopy images ofCΔcorA2,ΔcorA2, andΔcorA2 supplemented with exogenous Mg2+. Scale bar: 500 nm [file 13567_2026_1757_MOESM7_ESM.tif]

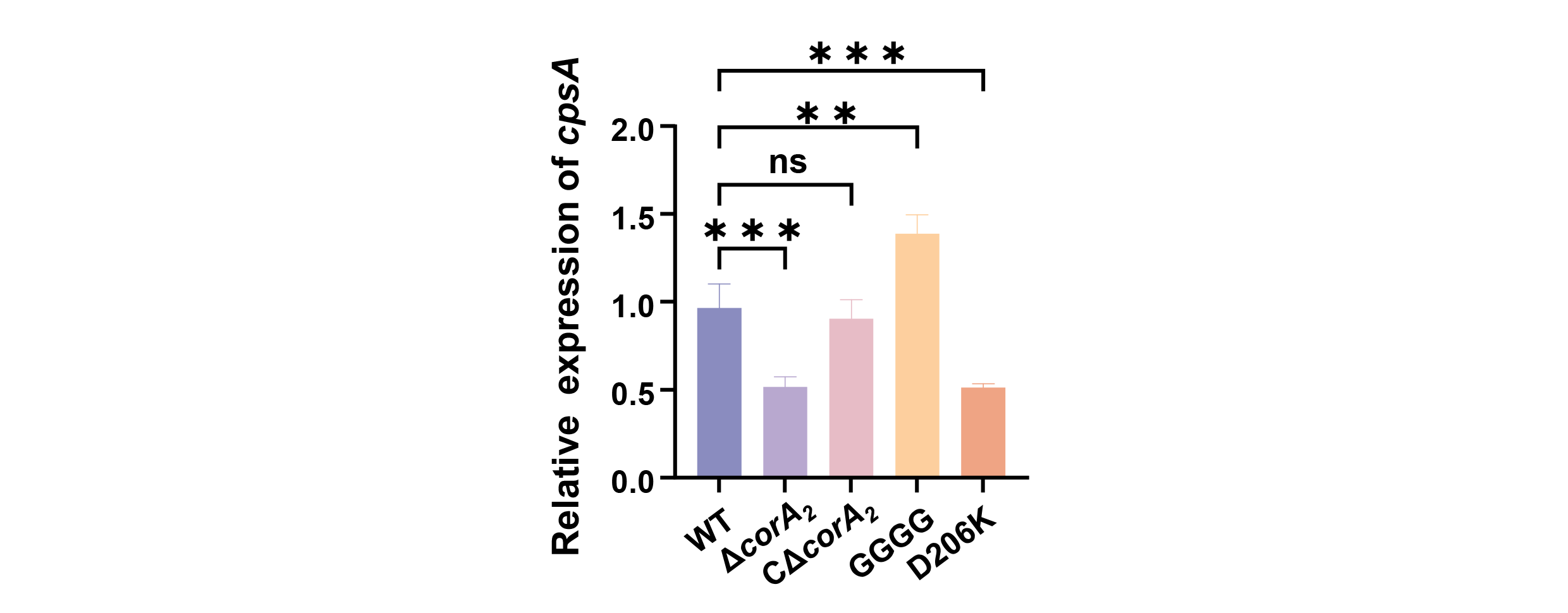

Supplement: Supplementary file 8 — Additional file 8: Expression analysis of the capsule synthesis key gene Acps. Transcriptional levels of cpsA in WT, ΔcorA2, CΔcorA2, GGGG, and D206K strains were determined by qRT-PCR. The 16S rRNA gene was used as an internal reference gene. All data are presented as mean ± SD from three independent biological replicates. Statistical significance was determined by one-way ANOVA. [file 13567_2026_1757_MOESM8_ESM.tif]
